# Multi-Objective Particle Swarm Optimization of Sensor Distribution Scheme with Consideration of the Accuracy and the Robustness for Deformation Reconstruction

**DOI:** 10.3390/s19061306

**Published:** 2019-03-15

**Authors:** Feifei Zhao, Hong Bao, Song Xue, Qian Xu

**Affiliations:** 1 Key Laboratory of Electronic Equipment Structure Design of Ministry of Education, Xidian University, Xi’an 710071, China; ffzhao@stu.xidian.edu.cn (F.Z.); sxue@xidian.edu.cn (S.X.); 2 Xinjiang Observatory, National Astronomical Observatories, Chinese Academy of Science, Urumqi 830011, China; xuqian@xao.ac.cn

**Keywords:** inverse finite element method, multi-objective particle swarm optimization, deformation reconstruction, transfer relationship, optimal model

## Abstract

For the inverse finite element method (iFEM), an inappropriate scheme of strain senor distribution would cause severe degradation of the deformation reconstruction accuracy. The robustness of the strain–displacement transfer relationship and the accuracy of reconstruction displacement are the two key factors of reconstruction accuracy. Previous research studies have been focused on single-objective optimization for the robustness of the strain–displacement transfer relationship. However, researchers found that it was difficult to reach a mutual balance between robustness and accuracy using single-objective optimization. In order to solve this problem, a bi-objective optimal model for the scheme of sensor distribution was proposed for this paper, where multi-objective particle swarm optimization (MOPSO) was employed to optimize the robustness and the accuracy. Initially, a hollow circular beam subjected to various loads was used as a case to perform the static analysis. Next, the optimization model was established and two different schemes of strain sensor were obtained correspondingly. Finally, the proposed schemes were successfully implemented in both the simulation calculation and the experiment test. It was found that the results from the proposed optimization model in this paper proved to be a promising tool for the selection of the scheme of strain sensor distribution.

## 1. Introduction

During the past few decades, deformation reconstruction has played an important role in real-time structural health monitoring (SHM) with the flourish of civil engineering, aerospace, and smart structure development. Shape sensing techniques can be employed to reconstruct the displacement field based on strain sensor values in a real-time manner [1,2]. The advantage of this method is that a priori knowledge, such as model shape, load form, and elastic-inertial material information, is not required in the process of reconstruction. This is particularly the case in scenarios where the applied loads are difficult to be accurately determined or measured. For example, the applied loads are the aerodynamic forces, temperatures, impact loads, or vibrating excitation [3].

The key technology in shape sensing was to accurately establish the mathematical model between the discrete surface strain and the displacement field. Previous researchers proposed many modeling methods. The adaptive fuzzy net was employed to establish the transformation relationship between measured surface strain and displacement [4]. Moreover, neural nets were also used to perform the strain–displacement relationship [5,6]. However, the shortcoming of these net approaches was that the reconstructed displacement accuracy was heavily dependent on the selection of the load cases used for the training process. Ko et al. performed the integration of discrete surface strain to handle the beam deformation problem [7]. Later, the shape sensing technique was further developed based on Ko’s displacement theory. This theory was then extended to solve the deformation displacement of beam, plate, and wing [8,9]. This method has shown a higher reconstructed accuracy in the resultant one-dimension than in the multi-dimensional loading conditions. The global or piecewise continuous basis functions have been used to describe the strain field [10,11,12,13,14,15,16]. In this paper, the authors have combined the spatial functions with the appropriate weight coefficient to fit the measured strains, where the unknown weights were determined by the discrete measurement of the strain. However, the required strain number was generally larger. When normal modes are employed as the basis functions, the deformation reconstructions are referred to as modal method (MM) [10,14]. Zhu et al discovered that the reconstruction accuracy of the modal method was higher than the neural net method, but the accuracy of the modal method relied on the number of modes used and it was hard to implement [15]. The discrete surface strains obtained by the strain sensors were interpolated into a predefined shape function to calculate the deformation displacement of the whole wing [16].

In the process of reconstructing the displacement field, the aforementioned approaches involved structural properties, load forms, and material parameters. However, in the case of application, it was difficult to determine the load information. In order to solve this problem, Tessler et al. proposed the inverse finite element method (iFEM), which employed the variational principle and performed the three-displacement field according to the strain–displacement relationship based on the Timoshenko beam theory [17,18]. Moreover, the first-order shear deformation theory (FSDT) was used to develop a three-node inverse shell element [19]. Additionally, some experiments have already been performed for the validation of the inverse finite element method [20,21].

During the application of the inverse finite element method, people have found that the schemes of strain sensor distribution had a great influence on the displacement reconstruction accuracy. The information entropy was proposed as a performance parameter of the sensor distribution. Then, the optimal sensor distribution schemes could be formulated as a single-objective optimization problem involving discrete-valued variables based on efficient sequential sensor placement algorithms [22]. Therefore, a single-objective optimal model for the scheme of sensor distribution could be presented to investigate how to maintain the stability of the section strain computing, where the optimal senor distribution scheme based on the particle swarm optimization could be obtained according to Reference [23,24]. For the aforementioned optimal methods, the single-objective optimal model was used to optimize the sensor distribution schemes to overcome the drawback of not fully considering reconstruction accuracy, which may degenerate other performances [23].

In order to further improve the performance using iFEM in the engineering application scenarios, the authors of this paper employed multi-objective particle swarm optimization (MOPSO) to optimize both the robustness and the accuracy simultaneously to obtain the optimal strain sensor distribution scheme. The paper is organized as follows. In the first section, a brief review of the inverse finite element method is presented. Next, bi-objective optimization functions are proposed based on the well-separated eigenvalues and the relative root mean square (RRMS). In the meantime, an introduction to the MOPSO is presented and the adopted optimization strategies are illustrated. Then, the optimization model is established considering both robustness and accuracy. Two different optimal schemes of strain sensor distribution are presented correspondingly. Finally, the two schemes are applied to a hollow circle beam subjected to various free-end loads. Both the simulation calculations and experiment tests were carried out. The experimental results showed that the optimization model established in this paper can effectively improve the performance of the inverse finite element method.

## 2. Inverse Finite Element Problem Specification

The original inverse finite element method (iFEM) was proposed in Reference [18], and developed continually by certain researchers. Academic achievements were obtained in Reference [17,18,19,20,21,22,23,24,25,26]. Therefore, in this section, the inverse finite element method for beam elements is briefly reviewed as follows.

Based on the small-strain hypothesis, the strains can be obtained from the deviation of displacement based on the Timoshenko theory along the x axes, as given by Equation (1):

(1) $$\begin{array}{l} \varepsilon_{x}\left(x,y,z\right)=e_{1}\left(x\right)+ze_{2}\left(x\right)+ye_{3}\left(x\right)\\ \gamma_{xz}\left(x,y\right)=e_{4}\left(x\right)+ye_{6}\left(x\right)\\ \gamma_{xy}\left(x,y\right)=e_{5}\left(x\right)-ze_{6}\left(x\right) \end{array}$$

where $e\left(u\right)=\left\{e_{1},e_{2},e_{3},e_{4},e_{5},e_{6}\right\}$ describes the section strain of the theory, and it can be presented further in terms of nodal displacement as follows:

(2) $$\begin{array}{l} e_{i}(u)=B_{i}u^{e}\\ u^{e}=[u,v,w,\theta_{x},\theta_{y},\theta_{z}] \end{array}$$

where $B_{i}$ (*i* = 1,…,6) is the deviation of the displacement shape function. $u^{e}$ contains the nodal displacement and rotation angle.

The iFEM uses the weighted least square principle to minimize the difference between in situ section strain, $e^{\varepsilon }$, and analytic section strains, e(u), which can be written as follows:

(3) $$\varphi \left(u\right)={\left\|e\left(u\right)-e^{\varepsilon }\right\|}^{2}$$

The relationship between in situ strain measurement and deformed beam displacement can be obtained by substituting Equation (2) into Equation (3), which can be expressed as follows:

(4) $$k^{e}u^{e}=f^{e}$$

The two terms $k^{e}$ and $f^{e}$ are defined as follows:

(5) $$k^{e}=\sum_{k=1}^{6}w_{k}k_{k}^{e}\;f^{e}=\sum_{k=1}^{6}w_{k}f_{k}^{e}$$

with

(6) $$k_{k}^{e}=\frac{L}{n}\sum_{i=1}^{n}\left[B_{k}^{T}\left(x_{i}\right)B_{k}\left(x_{i}\right)\right]\;f_{k}^{e}=\frac{L}{n}\sum_{i=1}^{n}\left[B_{k}^{T}\left(x_{i}\right)e_{k}^{\varepsilon }\left(x_{i}\right)\right]$$

where *n* denotes the section number, $x_{i}$ is the section location along the x axis, and $e_{i}^{\varepsilon }\left(x_{i}\right)$ is the in situ section strains. *L* denotes the element length.$w_{i}$ (*k* = 1,2,…,6) are positive-valued constants for the axial stretching, bending, twisting, and transverse shearing, respectively, and their initial values have been set as 1 in this paper.

**Figure 1:**
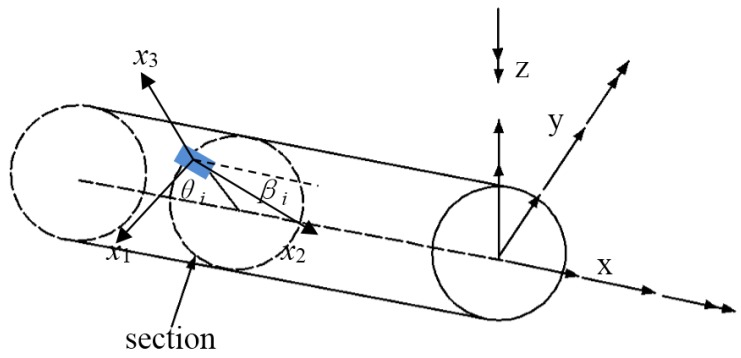
Location of the strain sensor placed on the beam external surface.

Once the scheme of strain sensor distribution has been determined, which means that the values of $x=x_{i},\theta =\theta_{i},\beta =\beta_{i}$ (*i* = 1,2,…,6) are determined, the section strains can be calculated from the measured surface strains using strain–tensor transformations as follows:

(7) $$\begin{array}{ll} \varepsilon_{2}^{*}\left(x_{i},\theta ,\beta \right)\;= & \left[\left(c_{\beta }^{2}-\mu s_{\beta }^{2}\right),\left(c_{\beta }^{2}-\mu s_{\beta }^{2}\right)s_{\theta }R,\left(c_{\beta }^{2}-\mu s_{\beta }^{2}\right)c_{\theta }R,c_{\beta }s_{\beta }c_{\theta },c_{\beta }s_{\beta }s_{\theta },c_{\beta }s_{\beta }R\right]\\ & \cdot \;{\left[e_{1}^{\varepsilon }\left(x_{i}\right),e_{2}^{\varepsilon }\left(x_{i}\right),e_{3}^{\varepsilon }\left(x_{i}\right),e_{4}^{\varepsilon }\left(x_{i}\right),e_{5}^{\varepsilon }\left(x_{i}\right),e_{6}^{\varepsilon }\left(x_{i}\right)\right]}^{T}\\ & =T\;\cdot \;{\left[e_{1}^{\varepsilon }\left(x_{i}\right),e_{2}^{\varepsilon }\left(x_{i}\right),e_{3}^{\varepsilon }\left(x_{i}\right),e_{4}^{\varepsilon }\left(x_{i}\right),e_{5}^{\varepsilon }\left(x_{i}\right),e_{6}^{\varepsilon }\left(x_{i}\right)\right]}^{T}\\ & \mathrm{with}\;c_{\beta }=\cos\beta ,c_{\theta }=\cos\theta \;s_{\beta }=\sin\beta \;\mathrm{and}\;s_{\theta }=\sin\theta , \end{array}$$

where μ is the Poisson ratio. *R* is the external radius of the section as shown in Figure 1.

Then, based on the analysis and calculation results of Equation (12) in Reference [23], these unknown parameters (*a*_1_, …, *a*_6_), which are used to established the relationship between section strains and the position along the centroid axis, can be solved:

(8) $${\left[a_{1},a_{2},a_{3},a_{4},a_{5},a_{6}\right]}^{T}={\left(T\times \left[\begin{array}{cccc} \begin{array}{c} 1\\ 0\\ 0 \end{array} & \begin{array}{c} 0\\ x_{i}\\ 0 \end{array} & \begin{array}{c} 0\\ 1\\ 0 \end{array} & \begin{array}{c} \begin{array}{ccc} 0 & 0 & 0 \end{array}\\ \begin{array}{ccc} 0 & 0 & 0 \end{array}\\ \begin{array}{ccc} x_{i} & 1 & 0 \end{array} \end{array}\\ 0 & \frac{D_{y}}{G_{z}} & 0 & \begin{array}{ccc} 0 & 0 & 0 \end{array}\\ 0 & 0 & 0 & \begin{array}{ccc} \frac{D_{z}}{G_{y}} & 0 & 0 \end{array}\\ 0 & 0 & 0 & \begin{array}{ccc} 0 & 0 & 1 \end{array} \end{array}\right]\right)}^{-1}\times \varepsilon_{2}^{*}\;={\left(T\times Q^{*}\right)}^{-1}\varepsilon_{2}^{*}$$

where $\varepsilon_{2}^{*}={\left[\varepsilon_{2}^{*}\left(x_{i},\theta_{i},\beta_{i}\right)\right]}^{T}$, $T={\left[T_{1},T_{2},\ldots ,T_{6}\right]}^{T}$, *i* = 1,2,…,6.

Therefore, for an arbitrary point on the centroid axis, the section strains (*e*_1_,*e*_2_,…*e*_6_) can be determined by substituting Equation (8) into Equation (7):

(9) $$\begin{array}{ll} {\left[e_{1}^{\varepsilon },e_{2}^{\varepsilon },e_{3}^{\varepsilon },e_{4}^{\varepsilon },e_{5}^{\varepsilon },e_{6}^{\varepsilon }\right]}^{T} & =Q\times {\left[a_{1},a_{2},a_{3},a_{4},a_{5},a_{6}\right]}^{T}\\ & =Q{\left(T\times Q^{*}\right)}^{-1}\varepsilon_{2}^{*} \end{array}$$

where the *Q* matrix can be obtained by replacing variable *x*_i_ by *y*_i_ in the $Q^{*}$ matrix, and *y*_i_ denotes an arbitrary point placement along the centroid axis direction.

The transfer relationship between deformation displacement and surface strain can be constructed by substituting Equation (9) into Equation (4). This relationship can be expressed as follows:

(10) $$u^{e}={\left(k^{e}\right)}^{-1}f^{e}=TR\times \varepsilon_{2}^{*}\left(x_{i}\right)$$

where $TR={\left(\frac{L}{n}\times \sum_{i=1}^{6}\left[w_{k}B_{k}^{T}\left(x_{i}\right)B_{k}\left(x_{i}\right)\right]\right)}^{-1}\times (\frac{L}{n}\times \sum_{i=1}^{6}\left[w_{k}B_{k}^{T}\left(x_{i}\right)Q{\left(T\times Q^{*}\right)}^{-1}\right])$.

## 3. Establishing the Multi-Objective Particle Swarm Optimization Model

The section strains can be computed from the strain sensor values which can be measured by Fiber Bragg Grating (FBG) sensors. However, during the FBG sensor installation, installation errors occur between the installation position and the theoretical position of the sensor along the axial length *x*_i_, angle parameter *θ*_i,_ and *β*_i_. Therefore, these position parameters with errors are substituted into Equation (7), which will affect the accuracy of the computed section strain. Moreover, in Equation (10), the transfer relationship (TR) is the matrix about the scheme of strain sensor distribution, and acts as a bridge between the surface strain and the deformation displacement. However, it was found that an inappropriate strain sensor distribution scheme could lead to a singular or ill-condition ***TR*** matrix [23]. For example, a tiny surface measured strain change might cause a significant reconstructed displacement change using the iFEM, which further deteriorates the accuracy or even leads to the process failure. Thus, the robustness of transfer relationship is one optimization objective ($f_{1}$), which can be constructed with well-separated eigenvalues [27]:

(11) $$\begin{array}{l} f\left(TR(\xi_{i},\theta_{i},\beta_{i})\right)=\max(\min\left|\lambda_{i}-\lambda_{j}\right|,i\neq j,i,j=1,2,\ldots ,6)\\ f_{1}=-f \end{array}$$

where $\lambda_{i}$ represents the eigenvalues of transformation matrix $TR(\xi_{i},\theta_{i},\beta_{i})$. Parameter $\xi_{i}$ represents the sensor location along the centroid axis direction. It is difficult to determine the strain sensor locations on both end nodes in engineering applications. Therefore, the installed placements are selected in the range of $\xi_{i}\in [\frac{L}{10},\frac{9L}{10}]$. In order to minimize the positioning errors for a strain sensor distribution scheme, one sensor is placed at $\beta_{i}=\;45^{{}^{\circ}}$, whereas the other five sensors are located at $\beta_{i}\;=\;0$ [23].

The accuracy is the key reference to estimate the performance of deformation reconstruction using the iFEM. Generally, structural beam deformation is varied due to different load forms. In order to eliminate the effect of working conditions on deformation, the relative root mean square (RRMS) is proposed to act as another optimization objective function ($f_{2}$) for estimating reconstruction accuracy, which can be expressed as follows:

(12) RMS=(∑i=1n(dispmeasurement(xi)−dispiFEM(xi))2/n2)f(RRMS(xi,θi,βi))=100%×RMSMax(dispmeasurement)f2=−f

where *disp*(*x*_i_) is the deformation displacement along the centroid axis in one direction. The superscript *measurement* denotes the actual deformation value from the simulation software or a measurement device. The *iFEM* is the predicted value computed from the iFEM. *RMS* indicates deformation reconstruction root-mean-square errors. *RRMS* is the ratio of RMS and maximum deformation value from the actual deformation value.

The purpose of strain sensor distribution scheme optimization is to find an optimal scheme, which can make the optimal balance between robustness and accuracy throughout a limited beam surface measurement. Thus, the optimization model can be described as follows:

(13) $$Minimize\;F\left(x_{i},\theta_{i},\beta_{i}\right)=\max\left[f_{1}\left(x_{i},\theta_{i},\beta_{i}\right),f_{2}\left(x_{i},\theta_{i},\beta_{i},\right)\right]\;S.T\;x_{i}\in [\frac{L}{10},\frac{9L}{10}],\theta_{i}\in [0{}^{\circ},360{}^{\circ}],\beta_{i}=0{}^{\circ},\;or,\;45{{}^{\circ}}^{}\;i=1,2,\ldots ,6.$$

For multiple objective optimization problems, Coello et al. proposed an extending particle swarm optimization (PSO) approach to solve this problem, referred to as an MOPSO [28]. Compared with other algorithms, the advantages of this method are a high convergence speed and a simple algorithm structure [29].

**Figure 2:**
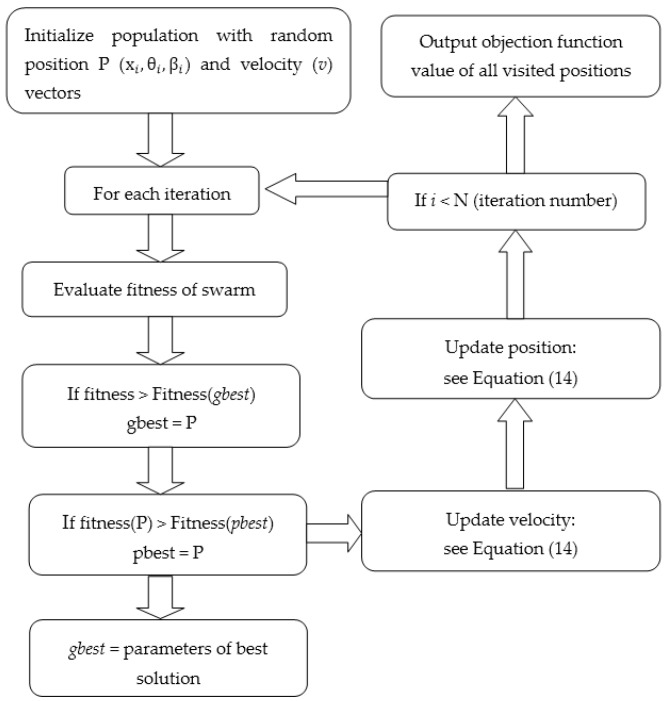
Flow chart depicting the multi-objective particle swarm optimization (MOPSO) algorithm.

According to the PSO algorithm theory [30,31,32], a population size is 50 and randomly initialized in the limited beam surface. The maximum iteration number is 100. Each particle position can be expressed by a vector *p_i_* = ($x_{i11}$, …, $x_{i16}$, … $x_{i31}$, …, $x_{i36}$, $\theta_{i11}$ …, $\theta_{i16}$, $\theta_{i31}$ …, $\theta_{i36}$)_1×36_, which are random initialized in *x_i_* ∈ [0,*L*], *θ_i_* ∈ [−180°, 180°], *β_i_* = 0° or 45°. Each particle has a corresponding velocity, represented by *v_i_* = ($v_{1x1}$, …, $v_{1x6}$, … $v_{3x1}$, …, $v_{3x6}$, $v_{1\theta 1}$ …, $v_{1\theta 6}$, $v_{3\theta 1}$ …, $v_{3\theta 6}$)_1×__36_, which determines the direction that particle can move for the search of the optimal solutions. Then, each particle is introduced into an optimal model to calculate a potential solution (13). The optimal solutions are continuously searched by updating the velocity and position of particle according to Equation (14):

(14) $$\begin{array}{l} x_{i}^{i+1}=x_{i}^{i}+v_{i}^{t+1}\\ v_{i}^{t+1}=w*v_{i}^{t}+c_{1}*r_{1}*(p_{i}-x_{i}^{t})+c_{1}*r_{2}*(p_{g}-x_{i}^{t}) \end{array}$$

where *t* indicates the *t*th iteration in the PSO algorithms. Parameter *w* denotes inertia weight, which is decreased from 0.9 to 0.4. Acceleration constant parameters are $c_{1}$ = $c_{2}$ = 2. Parameters $r_{1}$ and $r_{2}$ are random values uniformly distributed in [0,1]. Parameters $p_{i}$ and $p_{g}$ indicate the element of personal best solution (*pbest*) and global best solution(*gbest*). The maximum velocity ($v_{\max}$) is equal to the dynamic range in each dimension of the particle, and $v_{i}^{t+1}\in [-v_{\max},v_{\max}]$. PSO will be stopped if the *t*th iteration satisfies the predefined maximum number of iterations. The concrete specifications of this optimization algorithm are explained in Figure 2.

The *pbest* is continuously updated in the following process: in the search space, if a particle searches a new solution that dominates its *pbest*, the new solution can be selected as the new *pbest*. Otherwise, the old solution has a probability of 0.5 to be replaced by a new solution. The criterion of crowding distance [29], which is used to estimate the density of solutions surrounding a particular solution in the Pareto front, is proposed to select *gbest*. For example, when the value of crowding distance is large, the solution in the Pareto front will be deviated from its surrounding solutions. Additionally, the reflecting wall proposed in Reference [30] is used to improve the exploration of the particle.

## 4. Simulation Calculation and Experiment Verification

### 4.1. Simulation Calculation

**Figure 3:**
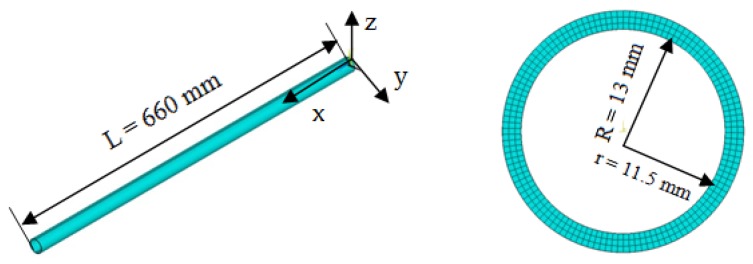
Finite element model of the cantilever beam.

In the simulation, the aluminum hollow beam using 188 elements was modeled based on the Timoshenko beam theory. The beam’s Young’s modulus is E = 73000 MPa with the Poisson ratio of v = 0.3 and a density of p = 2712.63 kg/m^3^. The beam length is L = 660 mm, with an external diameter R = 13 mm as well as an inner diameter r = 11.5 mm. The model was discreted into 200 elements and the cross-section of the beam was discreted into 120 segments as shown in Figure 3.

**Figure 4:**
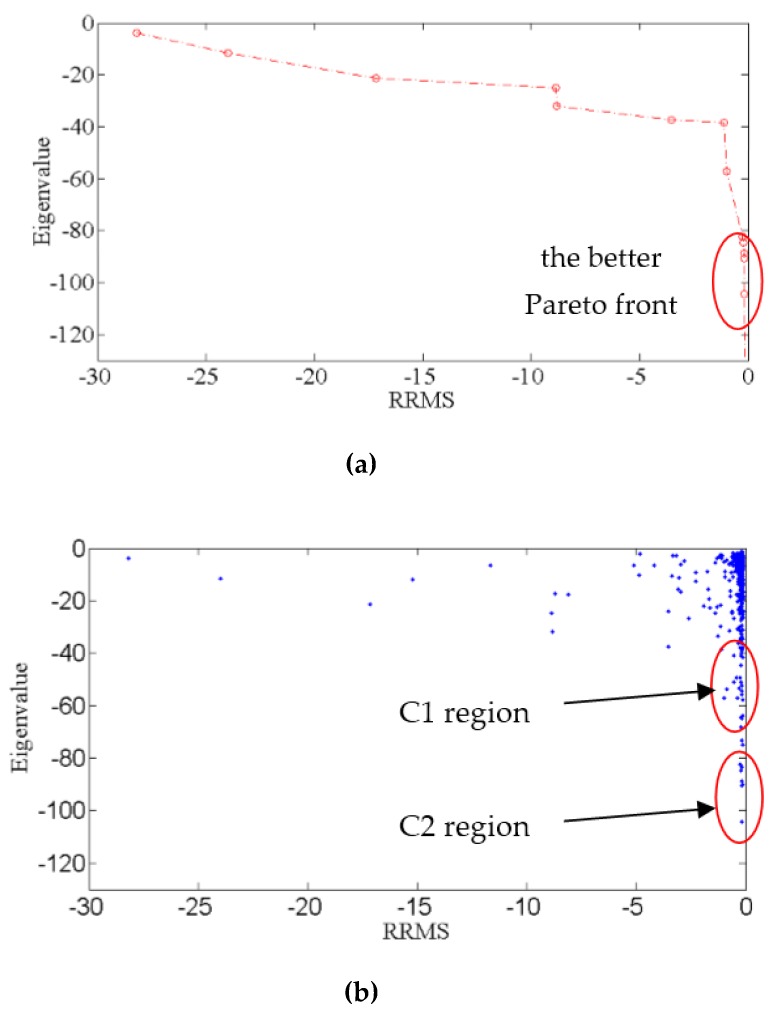
Optimal result: (**a**) Pareto front at the 100th iteration; (**b**) Objective function values of all positions visited by the swarm during optimization.

The free-end of the cantilever beam was subjected to static loads with a combination of two forces in three conditions (Table 1) for obtaining these parameters in nodes, containing deformation displacements, rotation angles, strains, and shear strains. Then, based on the obtained data, the MOPSO was established to optimize the strain sensor distribution scheme. The Pareto front and bi-objective function values ($f_{1},f_{2}$) are shown in Figure 4.

According to the Pareto front as shown in Figure 4a, the better front can be found in a small range. Figure 4b describes the objective function values of all positions visited by the swarm during optimization, which can be divided into the accurate region and the stable region, namely, C1 region and C2 region. However, in engineering, taking into consideration the strain senor distribution location errors, a good scheme should be searched in the C2 region, where a high accuracy and good robustness can be identified. On the other hand, the higher accuracy C1 region was also selected to compare the robustness of the C2 region. The specific strain sensor distribution schemes from ranges C1 and C2 are described in Table 2.

The C1 and C2 schemes were tested on the same hollow beam which was subjected to static loads with a two-force combination. The comparisons of iFEM deformation reconstruction of the hollow beam with two different schemes are presented in Table 3.

In this paper, the estimation criteria of reconstruction accuracy can be defined as follows:

(15) $$MER=MAX\left|disp{\left(x_{i}\right)}^{ANSYS}-disp{\left(x_{i}\right)}^{iFEM}\right|,i=1,\ldots 201,$$

where MER denotes the maximum errors. Max-disp is the maximum deformation displacement in theory. Table 3 shows that the reconstruction accuracy of the C1 scheme is higher than C2 on the condition of not considering the position errors of strain sensor distribution.

The robustness and the accuracy of the C1 and C2 schemes are further compared with the consideration of these errors from the strain sensor positions and measuring equipment, which may affect the accuracy of deformation reconstruction computed from Equation (10). It is assumed that these errors are as follows:

(16) $$\Delta x_{i}\in [-0.05,0.05],\;\Delta \theta_{i}\in \left[-9{}^{\circ},9{}^{\circ}\right]\;\mathrm{and}\;\Delta \beta_{i}\in [-10{}^{\circ},10{}^{\circ}],\;i=1,\ldots 6.$$

It is assumed that these noises are randomly distributed in the set, and these tests are performed 500 times in total. Then, the highest error can be selected for the reconstruction accuracy, which is shown in Table 4, Table 5.

Table 3 shows that the C1 scheme has a higher deformation reconstruction accuracy than C2 using iFEM without considering these disturbances. In particular, for the Y direction in heavy load, the error of the C2 scheme is 0.19 mm. On the other hand, the error of the C1 scheme drops to 0.08 mm.

When the noise is introduced, the maximum errors in the X, Y, Z directions are 1.01 mm, 20.6 mm, and 23.7 mm, respectively, for the C1 scheme. On the other hand, the maximum errors in the X, Y, Z directions drop to 2.64 mm, 2.61 mm, and 2.71 mm, respectively, for the C2 scheme, as shown in Table 4. The RRMS in the Y and Z directions are within 34% for the C1 scheme, and the RRMS in the Y and Z directions are stable within 4.8% for the C2 scheme, as shown in Table 5. It is indicated that the accuracy and the robustness of the C2 scheme is better than the C1 scheme.

### 4.2. Experiment Verification

**Figure 5:**
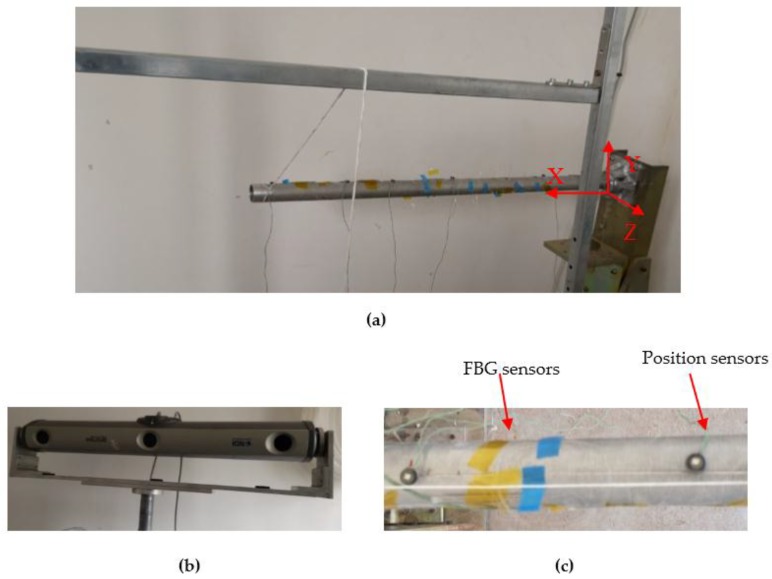
The component of the experimental platform: (**a**) A loading on the end node of the beam construction; (**b**) NDI Optrotrak Certus; (**c**) Position sensors and Fiber Bragg Grating (FBG) sensors.

Figure 5 shows the experiment rig and its measurement equipment, which includes a 3-D optical measurement device (NDI Optrotrak Certus, Northern Digital INC, Waterloo, ON, Canada) and Fiber Bragg Grating (FBG) strain sensors. The two-strain sensor distribution schemes and six-position sensors are used for the deformation reconstruction of the hollow beam under the end-node loadings, which are presented in Table 6.

In the experiments, the surface strains are measured by two schemes of the FBG strain sensor, respectively. In addition, the beam shapes are recorded by the NDI (in Figure 5b), which can accurately capture the position of the position sensors in Figure 5c. The comparison between the measured deformation captured from the NDI and the reconstructed deformation computed using the iFEM is shown in Table 7. Moreover, the deformation displacement values in measurement points are also shown in Table 8, Table 9, and Table 10.

From Table 7, MD^NDI^ denotes the maximum deformation measured by NDI. MDiFEMC1 and MDiFEMC2 denote the maximum deformation computed using the iFEM with the sensor distribution C1 and C2 schemes. MER_C1_ (MER_C2_) and RRMS_C1_ (RRMS_C2_) can be computed from Equations (17) and (20), respectively.

Table 7 compares the results of reconstructed displacement using the iFEM with the deformation displacement measured with NDI. It is found that the C2 scheme result gives a better robustness and a higher accuracy than the C1 scheme in most loading scenarios. In particular, the maximum errors (MER) in the Y and Z directions are 2.57 mm and 3.95 mm, and 0.2 mm and 0.16 mm, respectively, in most loading scenarios. The corresponding relative root mean square (RRMS) values are 22.8% and 20.9%, and 3.1% and 2.6%, respectively. However, in the X direction, the deformation displacement should be zero in theory, but there are slight deviations due to the imperfect distribution of the actual end-load in the YOZ plane. Under a heavy load, the deformation displacement of the hollow beam is only 0.49 mm, which gives the same magnitude order with the 3-D measurement device accuracy (NDI). Therefore, the maximum error and relative root mean square (RRMS) of reconstructed deformation displacement is comparatively high. Based on the aforementioned discussion, the optimal model proposed in this paper can allow the selection of a proper strain sensor distribution scheme which could result in a strain–displacement transfer relationship with better robustness and higher reconstructed deformation displacement accuracy.

## 5. Conclusions

The robustness of the strain–displacement relationship and the accuracy of the reconstructed deformation displacement are two key factors to evaluate the strain sensor distribution scheme. In this paper, the MOPSO was employed to reach a balance between robustness and accuracy. In the algorithm, the well-separated eigenvalues of transfer matrix was employed as an optimal objection, and the relative root mean square of reconstructed deformation displacement was used as another optimal objection. The optimization model was then tested on a hollow cantilever beam subjected to various loads. In the test, the Pareto front and the objective function values of all visited positions were obtained. Next, two schemes were chosen from the solution space based on practical application conditions and their results were compared. It was found that the C2 scheme could lead to a better strain–displacement relationship robustness as well as a higher reconstructed deformation displacement accuracy using the iFEM. Finally, the simulation results and experiment results with the disturbance errors added were used to validate the result that the C2 optimal scheme had a better robustness and a higher accuracy.

## Figures and Tables

**Table 1:** Loading types and directions on free-end.

	Heavy Load (N)	Middle Load (N)
Fy	250	120
Fz	200	100

**Table 2:** Optimized schemes of sensor distribution.

	C1	C2
$\varepsilon_{1}$	(−0.78, 25°, 0°)	(−0.54, −90°, 0°)
$\varepsilon_{2}$	(−0.36, 168°, 45°)	(−0.3, 150°, 0°)
$\varepsilon_{3}$	(−0.1, 66°, 0°)	(−0.24, −150°, 45°)
$\varepsilon_{4}$	(0.04, 69°, 0°)	(0.06, −80°, 0°)
$\varepsilon_{5}$	(0.46, 96°, 0°)	(0.28, 75°, 0°)
$\varepsilon_{6}$	(0.74, 93°, 0°)	(0.78, 45°, 0°)
*f*(*TR*)	22.8	94.5

**Table 3:** Comparison of reconstruction accuracy under two schemes (mm).

	Heavy Load (N)			Middle Load (N)			Slight Load (N)		
	X	Y	Z	X	Y	Z	X	Y	Z
*Max-disp*	0	37.8	30.3	0	18.2	15.1	0	7.57	7.57
*MER*									
C1	6.5 × 10^−4^	0.08	0.06	6.6× 10^−3^	0.07	0.07	3.7× 10^−4^	0.02	0.03
C2	8.5× 10^−4^	0.19	0.07	2.2× 10^−4^	0.11	0.09	2.01× 10^−4^	0.03	0.03

**Table 4:** Comparison of the maximum deformation errors with adding disturbance (mm).

	Heavy Load (N)			Middle Load (N)			Slight Load (N)		
	X	Y	Z	X	Y	Z	X	Y	Z
*Max*(*MER*)									
C1	1.01	20.60	23.70	0.47	9.62	10.96	0.21	4.22	4.80
C2	0.05	2.61	2.71	0.02	1.29	1.34	0.01	0.60	0.55

**Table 5:** Comparison of the maximum RRMS with adding disturbance (%).

	Heavy Load (N)			Middle Load (N)			Slight Load (N)		
	X	Y	Z	X	Y	Z	X	Y	Z
*RRMS*									
C1	0	24.48	33.92	0	23.04	30.61	0	23.73	26.11
C2	0	4.20	4.75	0	4.34	4.79	0	4.62	3.90

**Table 6:** Static loading case.

	Heavy Load (N)	Middle Load (N)	Slight Load (N)
Load (kg)	9	6	3

**Table 7:** Comparison between NDI and iFEM in the loading case of the end node.

	Heavy Load (N)			Middle Load (N)			Slight Load (N)		
	X (mm)	Y (mm)	Z (mm)	X (mm)	Y (mm)	Z (mm)	X (mm)	Y (mm)	Z (mm)
**MD^NDI^**	0.49	−5.13	−8.09	0.31	−3.13	−5.06	0.18	−1.73	−2.86
**MD^iFEMC1^**	0.27	−2.56	−4.14	0.23	−1.83	−2.72	0.15	−1.28	−1.81
**MD^iFEMC2^**	0.41	−5.00	−8.00	0.25	−3.07	−5.13	0.15	−1.81	−3.17
**MER_C1_**	0.22	2.57	3.95	0.23	1.31	2.34	0.15	0.45	1.25
**MER_C2_**	0.08	0.13	0.16	0.06	0.15	0.15	0.03	0.08	0.11
**RRMS_C1_**	61.2%	21.6%	20.9%	53.5%	17.6%	19.8%	52.9%	22.8%	18.6%
**RRMS_C2_**	13.1%	2.2%	1.8%	12.7%	2.6%	2.6%	12.7%	3.1%	2.4%

**Table 8:** Deformation displacement by NDI measurement and reconstruction with the C1 and C2 schemes in marker point for heavy load condition.

Measurement Point	NDI_X (mm)	NDI_Y (mm)	NDI_Z (mm)	C1_X (mm)	C1_Y (mm)	C1_Z (mm)	C2_X (mm)	C2_Y (mm)	C2_Z (mm)
1	0.08	−0.01	−0.16	0	0	0	0	0	0
2	0.2	−0.36	−0.72	0.06	−0.28	−0.42	0.15	−0.15	−0.56
3	0.25	−0.98	−1.50	0.14	−1.19	−1.81	0.22	−1.00	−1.34
4	0.35	−1.82	−2.79	0.18	−1.78	−2.76	0.28	−1.91	−2.64
5	0.35	−2.95	−4.45	0.22	−2.14	−3.38	0.34	−2.97	−4.30
6	0.49	−5.13	−8.09	0.27	−2.56	−4.14	0.41	−5.00	−8.00

**Table 9:** Deformation displacement by NDI measurement and reconstruction with the C1 and C2 schemes in marker point for middle load condition.

Measurement Point	NDI_X (mm)	NDI_Y (mm)	NDI_Z (mm)	C1_X (mm)	C1_Y (mm)	C1_Z (mm)	C2_X (mm)	C2_Y (mm)	C2_Z (mm)
1	0.05	−0.01	−0.13	0	0	0	0	0	0
2	0.12	−0.30	−0.49	0.04	−0.20	−0.57	0.09	−0.27	−0.36
3	0.15	−0.62	−1.02	0.09	−0.84	−1.18	0.14	−0.61	−0.86
4	0.22	−1.08	−1.81	0.12	−1.26	−1.80	0.18	−1.18	−1.69
5	0.22	−1.68	−2.91	0.14	−1.53	−2.21	0.21	−1.84	−2.76
6	0.31	−3.13	−5.06	0.17	−1.82	−2.72	0.25	−3.07	−5.13

**Table 10:** Deformation displacement by NDI measurement and reconstruction with the C1 and C2 scheme in marker point for slight load condition.

Measurement point	NDI_X (mm)	NDI_Y (mm)	NDI_Z (mm)	C1_X (mm)	C1_Y (mm)	C1_Z (mm)	C2_X (mm)	C2_Y (mm)	C2_Z (mm)
1	0.04	0	−0.08	0	0	0	0	0	0
2	0.08	−0.18	−0.3	0.03	−1.4	−0.18	0.06	−0.15	−0.23
3	0.08	−0.32	−0.61	0.06	−0.59	−0.77	0.08	−0.35	−0.53
4	0.13	−0.6	−1.08	0.08	−0.88	−1.18	0.11	−0.67	−1.05
5	0.13	−0.99	−1.74	0.09	−1.07	−1.46	0.13	−1.05	−1.71
6	0.18	−1.73	−2.86	0.11	−1.28	−1.81	0.15	−1.81	−3.17
